# Supplementary figures and images for: Increased entropy of signal transduction in the cancer metastasis phenotype
Source: BMC Syst Biol. 2010 Jul 30;4:104. doi: 10.1186/1752-0509-4-104 (PMC2925356; doi:10.1186/1752-0509-4-104)

**A)**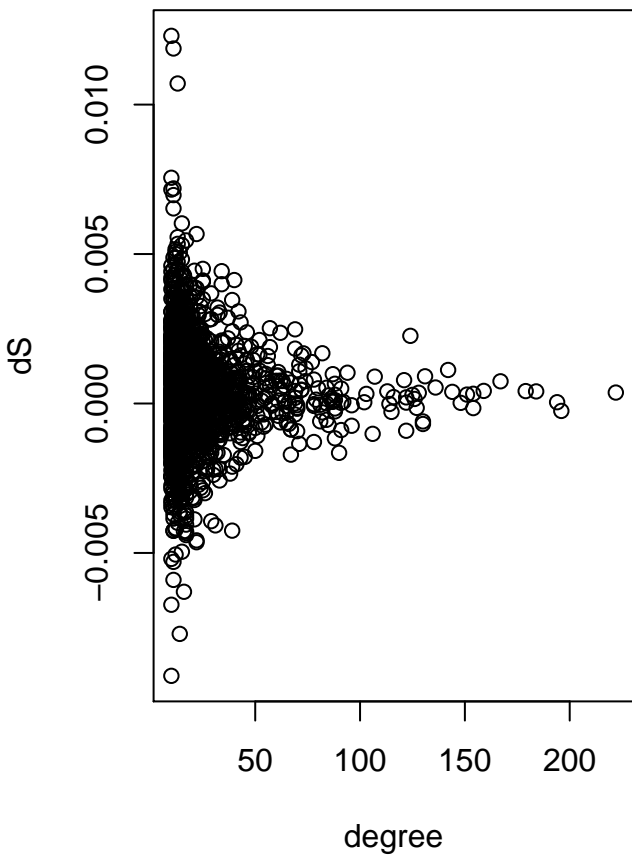**B)**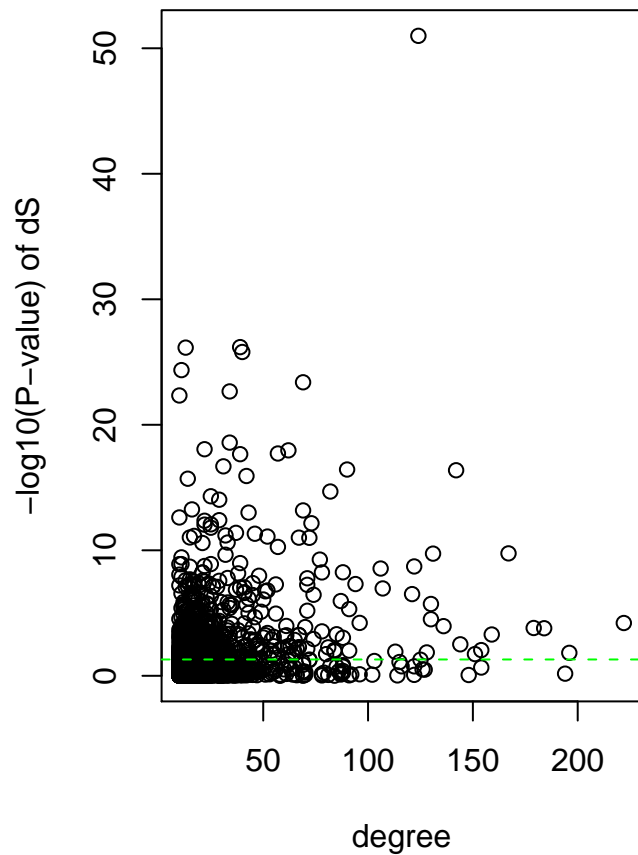

Supplement: Additional file 1 — Differential entropy and node degree. A) Differential entropy change is plotted against node degree. One observes a skew in the sense that high degree nodes exhibit smaller changes in entropy. This is a theoretical consequence of the entropy defintion. Thus using to rank genes is biased to nodes of low degree. B) The negatiive of the log(P-values) of differential entropies against node degree. Evaluating the statistical significance of the entropy changes we now observe that for every degree there are significant changes in entropy, thus removing the skew. Green line is line defined by P = 0.05. [file 1752-0509-4-104-S1.PDF]

**Significant entropy increases**

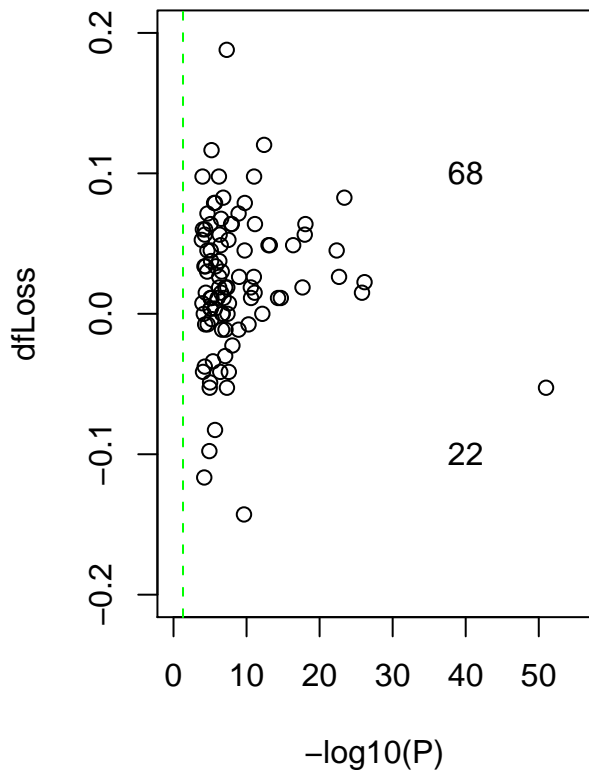

**No significant entropy change**

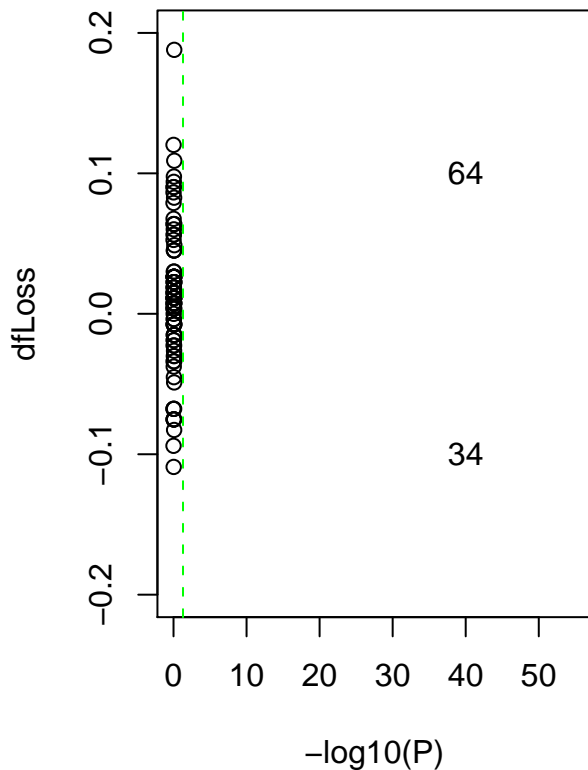

Supplement: Additional file 4 — Increased entropy and genomic loss. Of the top 200 nodes with most signficant entropy changes, 133 showed significant increases in the metastatic phenotype, and 90 of these genes could be mapped to an oligo array comparative genomic hybridisation study over 171 breast tumours [31]. Left panel plots the difference in the frequency of loss of the gene between the tumours that metastasized and those that did not (y-axis) against the negative logarithm of the P-value for the differential entropy (x-axis). Right panel shows the same plot for the 133 genes showing least significant changes in entropy. Genes with significant increases in entropy were 1.6 times more likely to be more frequently lost in poor prognosis tumours (Fisher test P = 0.08). [file 1752-0509-4-104-S4.PDF]
